# Gradient boosting machines, a tutorial

**DOI:** 10.3389/fnbot.2013.00021

**Published:** 2013-12-04

**Authors:** Alexey Natekin, Alois Knoll

**Affiliations:** ^1^fortiss GmbHMunich, Germany; ^2^Department of Informatics, Technical University MunichGarching, Munich, Germany

**Keywords:** boosting, gradient boosting, machine learning, regression, classification, robotic control, text classification

## Abstract

Gradient boosting machines are a family of powerful machine-learning techniques that have shown considerable success in a wide range of practical applications. They are highly customizable to the particular needs of the application, like being learned with respect to different loss functions. This article gives a tutorial introduction into the methodology of gradient boosting methods with a strong focus on machine learning aspects of modeling. A theoretical information is complemented with descriptive examples and illustrations which cover all the stages of the gradient boosting model design. Considerations on handling the model complexity are discussed. Three practical examples of gradient boosting applications are presented and comprehensively analyzed.

## 1. Introduction

A common task that appears in different machine learning applications is to build a non-parametric regression or classification model from the data. When designing a model in domain-specific areas, one strategy is to build a model from theory and adjust its parameters based on the observed data. Unfortunately, in most real-life situations such models are not available. In most situations even initial expert-driven guesses about the potential relationships between input variables are not available to the researcher. The lack of a model can be circumvented if one applies non-parametric machine learning techniques like neural networks, support vector machines, or any other algorithm at one's own discretion, to build a model directly from the data. These models are built in the supervised manner, which means that the data with the desired target variables has to be prepared beforehand.

The most frequent approach to data-driven modeling is to build only a single strong predictive model. A different approach would be to build a bucket, or an ensemble of models for some particular learning task. One can consider building a set of “strong” models like neural networks, which can be further combined altogether to produce a better prediction. However, in practice, the ensemble approach relies on combining a large number of relatively weak simple models to obtain a stronger ensemble prediction. The most prominent examples of such machine-learning ensemble techniques are random forests (Breiman, 2001) and neural network ensembles (Hansen and Salamon, 1990), which have found many successful applications in different domains (Liu et al., 2004; Shu and Burn, 2004; Fanelli et al., 2012; Qi, 2012).

The common ensemble techniques like random forests rely on simple averaging of models in the ensemble. The family of boosting methods is based on a different, constructive strategy of ensemble formation. The main idea of boosting is to add new models to the ensemble sequentially. At each particular iteration, a new weak, base-learner model is trained with respect to the error of the whole ensemble learnt so far. The first prominent boosting techniques were purely algorithm-driven, which made the detailed analysis of their properties and performance rather difficult (Schapire, 2002). This led to a number of speculations as to why these algorithms either outperformed every other method, or on the contrary, were inapplicable due to severe overfitting (Sewell, 2011).

To establish a connection with the statistical framework, a gradient-descent based formulation of boosting methods was derived (Freund and Schapire, 1997; Friedman et al., 2000; Friedman, 2001). This formulation of boosting methods and the corresponding models were called the gradient boosting machines. This framework also provided the essential justifications of the model hyperparameters and established the methodological base for further gradient boosting model development.

In gradient boosting machines, or simply, GBMs, the learning procedure consecutively fits new models to provide a more accurate estimate of the response variable. The principle idea behind this algorithm is to construct the new base-learners to be maximally correlated with the negative gradient of the loss function, associated with the whole ensemble. The loss functions applied can be arbitrary, but to give a better intuition, if the error function is the classic squared-error loss, the learning procedure would result in consecutive error-fitting. In general, the choice of the loss function is up to the researcher, with both a rich variety of loss functions derived so far and with the possibility of implementing one's own task-specific loss.

This high flexibility makes the GBMs highly customizable to any particular data-driven task. It introduces a lot of freedom into the model design thus making the choice of the most appropriate loss function a matter of trial and error. However, boosting algorithms are relatively simple to implement, which allows one to experiment with different model designs. Moreover the GBMs have shown considerable success in not only practical applications, but also in various machine-learning and data-mining challenges (Bissacco et al., 2007; Hutchinson et al., 2011; Pittman and Brown, 2011; Johnson and Zhang, 2012).

From the viewpoint of Neurorobotics, ensemble models are a useful practical tool for different predictive tasks, as they can consistently provide higher accuracy results compared to conventional single strong machine learning models. For example, the ensemble models can efficiently map the EMG and EEG sensor readings to human movement tracking and activity recognition. However, these models can also provide valuable insights into the models of neural formation and memory simulations. Whilst artificial neural networks have the memory of the learned patterns distributed within the connections of artificial neurons, in boosted ensembles the base-learners play the role of the memory medium and are forming the captured patterns sequantially, gradually increasing the level of pattern detail. Advances in boosted ensembles can find fruitful applications in the brain simulation domain, as the ensemble formation models can be coupled with the strategies of network growth. In particular, if the base-learners are considered the nodes of the network, which in the context of connectome will mean the neurons, it will be possible to construct ensembles with various graph properties and topologies, like small-world networks, which are found in the biological neural networks. In order to proceed with advanced neurorobotics applications of boosted ensemble models, it is essential to first define the methodology and algorithmic framework for these models.

In this article, we would provide the newcomers to the GBMs with both the formal description of the method and with considerations for the model design, which are illustrated on a number of practical examples. The article has a strong focus on machine learning aspects of GBM modeling, therefore the methodology section of the article is intended to readers with the appropriate statistical background. In section II, we describe the boosting methodology and the gradient boosting algorithm in detail. In section III, we discuss the GBM design opportunities. In section IV, regularization issues are concerned with a deeper insight into the dependencies between the model hyperparameter presented. section V provides the considerations for the model interpretation. In section VI, the application examples of GBMs are presented. In section VII, the overall GBM discussion and open issues are given, which are followed by conclusions in section VIII.

## 2. Methodology

In this section we present the basic methodology and learning algorithms of the GBMs, as originally derived by Friedman (2001). The tutorial is considered an introduction to the GBMs, therefore the strict mathematical proofs of algorithms and their properties are not covered in this article.

### 2.1. Function estimation

Consider the problem of function estimation in the classical supervised learning setting. The fact that the learning is supervised leaves a strong restriction on the researcher, as the data has to be provided with the sufficient set of proper target labeles (which can be very costly to extract, e.g., come form an expensive experiment). We arrive with the dataset (*x*, *y*)^*N*^_*i* = 1_, where *x* = (*x*_1_, …, *x*_*d*_) refers to the explanatory input variables and *y* to the corresponding labels of the response variable. The goal is to reconstruct the unknown functional dependence $x\overset{f}{\to }y$ with our estimate $\hat{f}(x)$, such that some specified loss function Ψ(*y, f*) is minimized:

(1) $$\begin{array}{l} \;\hat{f}(x)=y,\\ \hat{f}(x)=\arg\underset{f(x)}{\min}\Psi (y,f(x)) \end{array}$$

Please note that at this stage, we don't make any assumptions about the form of neither the true functional dependence *f*(*x*), nor the form of the function estimate $\hat{f}(x)$. If we rewrite the estimation problem in terms of expectations, the equivalent formulation would be to minimize the expected loss function over the response variable *E*_*y*_(Ψ[*y, f*(*x*)]), conditioned on the observed explanatory data *x*:

(2) $$\hat{f}(x)=\arg\underset{f(x)}{\min}\underset{\text{expectation over the whole dataset}}{\underset{︸}{E_{x}[\overset{\text{expected y loss}}{\overset{︷}{E_{y}(\Psi [y,f(x)])}}|x]}}$$

The response variable *y* can come from different distributions. This naturally leads to specification of different loss functions Ψ. In particular, if the response variable is binary, i.e., *y* ∈ {0, 1}, one can consider the binomial loss function. If the response variable is continuous, i.e., *y* ∈ *R*, one can use classical *L*_2_ squared loss function or the robust regression Huber loss. For other response distribution families like the Poisson-counts, specific loss functions have to be designed. More details on the types of loss functions are presented in the III section of the article.

To make the problem of function estimating tractable, we can restrict the function search space to a parametric family of functions *f*(*x*, θ). This would change the function optimization problem into the parameter estimation one:

(3) $$\hat{f}(x)=f(x,\hat{\theta }),$$

(4) $$\hat{\theta }=\arg\underset{\theta }{\min}E_{x}\left[E_{y}\left(\Psi \left[y,f(x,\theta )\right]\right)|x\right]$$

Typically the closed-form solutions for the parameter estimates are not available. To perform the estimation, iterative numerical procedures are considered.

### 2.2. Numerical optimization

Given *M* iteration steps, the parameter estimates can be written in the incremental form:

(5) $$\hat{\theta }=\sum_{i=1}^{M}{\hat{\theta }}_{i}$$

The simplest and the most frequently used parameter estimation procedure is the steepest gradient descent. Given *N* data points (*x, y*)^*N*^_*i* = 1_ we want to decrease the empirical loss function *J*(θ) over this observed data:

(6) $$J(\theta )=\sum_{i=1}^{N}\Psi (y_{i},f(x_{i},\hat{\theta }))$$

The classical steepest descent optimization procedure is based on consecutive improvements along the direction of the gradient of the loss function ∇*J*(θ). As the parameter estimates $\hat{\theta }$ are presented in an incremental way, we would distinguish the estimate notation. By the subscript index of the estimates ${\hat{\theta }}_{t}$ we would consider the *t*-th incremental step of the estimate $\hat{\theta }$. The superscript ${\hat{\theta }}^{t}$ corresponds to the collapsed estimate of the whole ensemble, i.e., sum of all the estimate increments from step 1 up till step *t*. The steepest descent optimization procedure is organized as follows:

1. Initialize the parameter estimates ${\hat{\theta }}_{0}$
  For each iteration *t*, repeat:
2. Obtain a compiled parameter estimate ${\hat{\theta }}^{t}$ from all of the previous iterations:
  (7) $${\hat{\theta }}^{t}=\sum_{i=0}^{t-1}{\hat{\theta }}_{i}$$
3. Evaluate the gradient of the loss function ∇*J*(θ), given the obtained parameter estimates of the ensemble:
  (8) $$\nabla J(\theta )=\{\nabla J(\theta_{i})\}={\left[\frac{\partial J(\theta )}{\partial J(\theta_{i})}\right]}_{\theta ={\hat{\theta }}^{t}}$$
4. Calculate the new incremental parameter estimate ${\hat{\theta }}_{t}$:
  (9) $${\hat{\theta }}_{t}\leftarrow -\nabla J(\theta )$$
5. Add the new estimate ${\hat{\theta }}_{t}$ to the ensemble

### 2.3. Optimization in function space

The principle difference between boosting methods and conventional machine-learning techniques is that optimization is held out in the function space. That is, we parameterize the function estimate $\hat{f}$ in the additive functional form:

(10) $$\hat{f}(x)={\hat{f}}^{M}(x)=\sum_{i=0}^{M}{\hat{f}}_{i}(x)$$

In this representation, *M* is the number of iterations, ${\hat{f}}_{0}$ is the initial guess and ${\left\{{\hat{f}}_{i}\right\}}_{i=1}^{M}$ are the function increments, also called as “boosts.”

To make the functional approach feasible in practice, one can follow a similar strategy of parameterizing the family of functions. Here we introduce to the reader the parameterized “base-learner” functions *h*(*x*, θ) to distinguish them from the overall ensemble function estimates $\hat{f}(x)$. One can choose different families of base-learners such as decision trees or splines. Various choices of base-learner models are considered and described in the appropriate section of this article.

We can now formulate the “greedy stagewise” approach of function incrementing with the base-learners. For this purpose the optimal step-size ρ should be specified at each iteration. For the function estimate at the *t*-th iteration, the optimization rule is therefore defined as:

(11) $${\hat{f}}_{t}\leftarrow {\hat{f}}_{t-1}+\rho_{t}h(x,\theta_{t})$$

(12) $$\left(\rho_{t},\theta_{t}\right)=\arg\min_{\rho ,\theta }\sum_{i=1}^{N}\Psi (y_{i},{\hat{f}}_{t-1})+\rho h\left(x_{i},\theta \right)$$

### 2.4. Gradient boost algorithm

One can arbitrarily specify both the loss function and the base-learner models on demand. In practice, given some specific loss function Ψ(*y, f*) and/or a custom base-learner *h*(*x*, θ), the solution to the parameter estimates can be difficult to obtain. To deal with this, it was proposed to choose a new function *h*(*x*, θ_*t*_) to be the most parallel to the negative gradient {*g*_*t*_(*x*_*i*_)}^*N*^_*i* = 1_ along the observed data:

(13) $$g_{t}(x)=E_{y}{\left[\frac{\partial \Psi (y,f(x))}{\partial f(x)}|x\right]}_{f(x)={\hat{f}}^{t-1}(x)}$$

Instead of looking for the general solution for the boost increment in the function space, one can simply choose the new function increment to be the most correlated with −*g*_*t*_(*x*). This permits the replacement of a potentially very hard optimization task with the classic least-squares minimization one:

(14) $$\left(\rho_{t},\theta_{t}\right)=\arg\min_{\rho ,\;\theta }\sum_{i=1}^{N}{\left[-g_{t}(x_{i})+\rho h(x_{i},\theta )\right]}^{2}$$

To summarize, we can formulate the complete form of the gradient boosting algorithm, as originally proposed by Friedman (2001). The exact form of the derived algorithm with all the corresponding formulas will heavily depend on the design choices of Ψ(*y, f*) and *h*(*x*, θ). One can find some common examples of these algorithms in Friedman (2001).

If we consider connections to earlier developments, it will turn out that the well known cascade correlation neural networks (Fahlman and Lebiere, 1989; Yao, 1993) can be considered a special type of a gradient boosted model, as defined in Algorithm 1. Since the input-side weights of each neuron become fixed right after it was added to the network, this whole model can be considered a GBM, where the base-learner model is just one neuron and the loss function is the standard squared error. This algorithm also maximizes the correlation between the error of the whole network and the newly created neuron, which makes the comparison more evident.

**Algorithm 1:** Friedman's Gradient Boost algorithm.

**Inputs:** input data (*x*, *y*)^*N*^_*i* = 1_ number of iterations *M* choice of the loss-function Ψ(*y*, *f*) choice of the base-learner model *h*(*x*, θ)
**Algorithm:** 1: initialize ${\hat{f}}_{0}$ with a constant 2: **for** *t* = 1 to *M* **do** 3: compute the negative gradient *g*_*t*_(*x*) 4: fit a new base-learner function *h*(*x*, θ_*t*_) 5: find the best gradient descent step-size ρ_*t*_: $$\rho_{t}=\arg\min_{\rho }\sum_{i=1}^{N}\Psi \left[y_{i},\;{\hat{f}}_{t-1}(x_{i})+\rho h(x_{i},\;\theta_{t})\right]$$ 6: update the function estimate: $${\hat{f}}_{t}\leftarrow {\hat{f}}_{t-1}+\rho_{t}h(x,\;\theta_{t})$$ 7: **end for**

## 3. GBM design

To design a particular GBM for a given task, one has to provide the choices of functional parameters Ψ(*y, f*) and *h*(*x*, θ). In other words, one has to specify what one is actually going to optimize, and afterwards, to choose the form of the function, which will be used in building the solution. It is clear that these choices would greatly affect the GBM model properties. The GBM framework provides the practitioner with such design flexibility.

This section provides the descriptions and illustrations of different families of loss functions and models of base-learners. For additional information on derivation and properties of the particular component of the GBM model please follow the corresponding references.

### 3.1. Loss-function families

Given a particular learning task, one can consider different loss functions Ψ(*y, f*) to exploit. This choice is often influenced by the demand of specific characteristics of the conditional distribution. The most frequent examples of such property is the robustness to outliers, but other opportunities can also be considered.

To use an arbitrary loss function, one has to specify both the loss function and the function to calculate the corresponding negative gradient. Given these two functions, they can be directly substituted into the GBM algorithm. In practice, many of the loss functions have already been derived for the GBM algorithm (Friedman, 2001; Schmid and Hothorn, 2008; Schmid et al., 2011).

Loss-functions can be classified according to the type of response variable *y*. Specific boosting algorithms have been derived for various families of the response, among which are the regression, classification and time-to-event analysis tasks. Depending on the family of response variable *y* we can systemize the most frequently used loss-functions as follows:

1. Continuous response, *y* ∈ *R*:
  - Gaussian *L*_2_ loss function
  - Laplace *L*_1_ loss function
  - Huber loss function, δ specified
  - Quantile loss function, α specified
2. Categorical response, *y* ∈ {0, 1}:
  - Binomial loss function
  - Adaboost loss function
3. Other families of response variable:
  - Loss functions for survival models
  - Loss functions counts data
  - Custom loss functions

To provide a better insight into the model design, we will describe the loss-functions for continuous and categorical response variables in more detail. Specific GBM algorithms have also been derived for other types of response like the Poisson-counts and the survival data, but we will not address these models in this paper.

#### 3.1.1. Loss functions for continuous response

When the response variable *y* is continuous, a regression task is solved. A classic loss function, which is commonly used in practice is the squared-error *L*_2_ loss:

(15) $$\Psi {\left(y,f\right)}_{L_{2}}=\frac{1}{2}{\left(y-f\right)}^{2}$$

**Figure 1:**
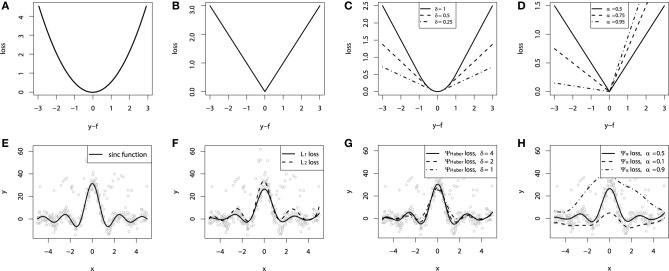
**Continuous loss functions: (A) *L*_2_ squared loss function; (B) *L*_1_ absolute loss function; (C) Huber loss function; (D) Quantile loss function**. Demonstration of fitting a smooth GBM to a noisy sinc(*x*) data: **(E)** original sinc(*x*) function; **(F)** smooth GBM fitted with *L*_2_ and *L*_1_ loss; **(G)** smooth GBM fitted with Huber loss with δ = {4, 2, 1}; **(H)** smooth GBM fitted with Quantile loss with α = {0.5, 0.1, 0.9}.

In the case of the *L*_2_ loss-function, its derivative is the residual *y* − *f*, which implies that the GBM algorithm simply performs residual refitting. The idea behind this loss function is to penalize large deviations from the target outputs while neglecting small residuals. The illustration of this loss function is provided on Figure 1A.

Another example is the absolute *L*_1_-loss, denoted as the “Laplacian” loss function. The *L*_1_-loss corresponds to the median of the conditional distribution, thus considered as the robust regression loss. The *L*_1_ loss function takes the form:

(16) $$\Psi {\left(y,f\right)}_{L_{1}}=|y-f|$$

It may be of particular interest in tasks where the response variable has long-tail error distribution. The function is illustrated on Figure 1B.

One can also exploit the parameterized loss-functions as well. A robust regression alternative to the *L*_1_ loss is the Huber loss function. It comprises two parts, corresponding to the *L*_2_ and *L*_1_ losses. The Huber loss is designed as follows:

(17) $$\Psi {\left(y,f\right)}_{\text{Huber},\delta }=\{\begin{array}{ll} \frac{1}{2}{\left(y-f\right)}^{2} & |y-f|\leq \delta \\ \delta (|y-f|-\delta /2) & |y-f|>\delta \end{array}$$

The cutting edge parameter δ is used to specify the robustification effect of the loss-function. The intuition behind this parameter is to specify the maximum value of error, after which the *L*_1_ loss has to be applied. The Huber loss function is illustrated on Figure 1C.

A more general approach is based on predicting a conditional quantile of the response variable (Koenker and Hallock, 2001). This approach is distribution free and in general proves to provide good robustness to outliers. The quantile loss is organized as follows:

(18) $$\Psi {\left(y,f\right)}_{\alpha }=\{\begin{array}{ll} \left(1-\alpha )|y-f\right| & y-f\leq 0\\ \alpha |y-f| & y-f>0 \end{array}$$

The parameter α in this case specifies the desired quantile of the conditional distribution. One can note that when α = 0.5, this would coincide with the *L*_1_ loss, thus resulting in the conditional median. Different parameterizations of the quantile loss function are illustrated on Figure 1D.

To demonstrate the properties of the described loss functions we will consider an artificially generated dataset. The dataset is sampled from a sinc(*x*) function with two sources of artificially simulated noise: the gaussian noise component ε ~ *N*(0, σ^2^) and the impulsive noise component ξ ~ Bern(*p*). The impulsive noise term is added to illustrate the robustification effects. The generated dataset is illustrated on Figure 1E.

To keep the experiment focused on the loss-function specifics we would assume that the learning was done in an optimal way. In this experiment the base-learner functions applied were the P-splines. The resulting GBM models of this experiment are presented on Figures 1F–H.

One can note that the median of the distribution is less affected by the impulsive noise whereas the *L*_2_ loss function is slightly biased due to the caused deviations. The quantile losses in their turn give a good estimation of the corresponding conditional distribution quantiles.

Following the idea of applying various loss-functions, one can for example model the conditional box-plots. From the computational perspective, this type of modeling would only result in increasing the number of different GBM models built by the number of desired statistics of the conditional distribution. However, it must be kept in mind that the resulting confidence intervals are a model approximation rather than true statistics. It is also important to note that the learned quantile models do not have to be necessary consistent with each other, as they are all learned separately.

#### 3.1.2. Loss functions for categorical response

In the case of categorical response, the response variable *y* typically takes on binary values *y* ∈ {0, 1}, thus, assuming that it comes from the Bernoulli distribution. To simplify the notation, let us assume the transformed labels *y*, putting *y* = 2*y* − 1 and making *y* ∈ {−1, 1}. In this case, the probability of class-wise response can be estimated by minimizing the negative log-likelihood, associated with the new class labels:

(19) $$\Psi {\left(y,f\right)}_{\text{Bern}}=\text{log}(1+\text{exp}(-2\bar{y}f))$$

**Figure 2:**
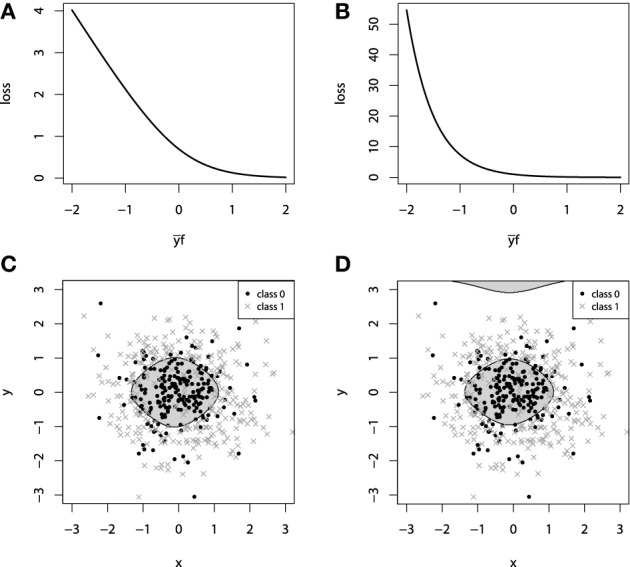
**(A)** Bernoulli loss function. **(B)** Adaboost loss function. **(C)** GBM 2d classification with Bernoulli loss. **(D)** GBM 2d classification with Adaboost loss.

This loss function is commonly referred to as the Bernoulli loss. The illustration of the Bernoulli loss function is given on Figure 2A. The chart shows the loss function defined over the values of *y**f*. Please note, that in this notation, positive values of *y**f* correspond to the correct discrimination.

Another common choice of categorical loss-function is the simple exponential loss, as it is used in the Adaboost algorithm (Schapire, 2002). Following the same notation as in the Bernoulli loss, the Adaboost loss function is therefore defined as:

(20) $$\Psi {\left(y,f\right)}_{\text{Ada}}=\text{exp}(-\bar{y}f)$$

It is possible to establish a connection between the influence trimming of GBMs with the Adaboost loss function and weight trimming Adaboost algorithm (Friedman, 2001). The illustration of the Adaboost loss is given on Figure 2B. The notation for this loss-function chart is the same as we used in the figure with Bernoulli loss.

To demonstrate the properties of the categorical loss functions we will construct another artificial dataset. Originally, all the data comes from a 2-dimensional normal distribution, with zero mean and identity-covariance matrix. The points that lie within the inner circle of unit radius *r* = 1 belong to one class and are colored with black, whereas all the other points are assigned to another class, colored with dark gray. For this setting, we use two sources of noise: 2-dimensional gaussian noise (ε_1_, ε_2_), ε_*i*_ ~ *N*(0, 0.3^2^) and a random misclassification error ξ, which randomly switches the class. The random misclassification results in slightly heavier tails of the distances of class-error distributions and thus, will allow us to contrast the difference between loss functions. The resulting dataset together with the marginal density plots of each class is presented on Figures 2C,D.

For both of the inspected models on Figure 2, the model complexity was chosen equally in terms of the number of boosting iterations *M*. It is, therefore, interesting to note that the models achieved similar accuracy, with equal confusion matrices. However, despite these two similarities, geometrically these models are considerably different. Due to the fact that the exponential loss of Adaboost model contrasts misclassified points much more, the corresponding model began capturing the boundary, “far”-outlying points, much earlier than the other model.

In the context of loss-functions, we say “much earlier” because it is true that at some point in the learning process we can overestimate the model-complexity and thus overfit the data with both types of loss functions. However, due to the nearly linear impact of outliers to the Bernoulli loss, the Bernoulli model is typically less-sensitive to this type of erroneous labeling in the data.

### 3.2. Specifying the base-learners

A particular GBM can be designed with different base-learner models on board. A diverse set of base-learners have been introduced in the literature thus far. In this subsection, we will briefly describe and illustrate the base-learner models that are most frequently used in practice.

The commonly used base-learner models can be classified into a three distinct categories: linear models, smooth models and decision trees. There is also a number of other models, such as markov random fields (Dietterich et al., 2004) or wavelets (Viola and Jones, 2001), but their application arises for relatively specific practical tasks. The base-learner model systematization with the corresponding examples of functions is organized as follows:

1. Linear models:
  - Ordinary linear regression
  - Ridge penalized linear regression
  - Random effects
2. Smooth models:
  - P-splines
  - Radial basis functions
3. Decision trees
  - Decision tree stumps
  - Decision trees with arbitrary interaction depth
4. Other models:
  - Markov Random Fields
  - Wavelets
  - Custom base-learner functions

An important design opportunity is that nothing prevents the practitioner from specifying a complex model, utilizing several classes of base-learner models in one GBM. This means that the same functional formula can include both smooth additive components and the decision trees components at the same time. Another example would be to split the explanatory variables into the categorical and smooth subspaces and fit different boosted base-learner models to each of the subspaces simultaneously.

Another important feature of the base-learner specification is that they can be designed for different models of variable interactions. If we consider the ANOVA decomposition of the function estimate $\hat{f}$, different interaction terms would correspond to the different interrelationships between explanatory variables:

(21) $$\hat{f}(x)=\sum_{j}f_{j}(x_{j})+\sum_{jk}f_{jk}(x_{jk})+\sum_{jkl}f_{jk}(x_{jkl})+\ldots$$

#### 3.2.1. Additive base-learners

Using the additive base-learner models explicitly assumes that there is no interaction between the explanatory variables. Yet, there has been mounting empirical evidence that for most practical tasks, simple additive models corresponding to the first term of the ANOVA decomposition, provide considerably accurate results (Schapire, 2002; Wenxin, 2002). Another important observation is that the resulting additive models are interpretable by design, allowing the practitioner to investigate each of the model components separately.

The learning algorithm for additive GBM models slightly differs from the algorithm we described earlier. At each iteration, several additive base-learner candidates, built atop some randomly chosen variables, are fitted simultaneously. Next, the best of these models is chosen, based on the residual sum of squares criterion. One property of this learning process is that it often leads to the situation, when many of the explanatory variables are omitted, thus, naturally leading to a sparser solution.

Motivation for using the additive representation with linear and generalized linear models (GLM) instead of the common GLM model with some penalty, is particularly based on the desire to fit a sparse model. This becomes especially important in tasks with many categorical variables, like the data that comes from medical and biological experiments. The resulting gradient boosting fitting leads to a relatively easy variable-selection procedure by design.

The choice of boosting the additive models is also sometimes dictated by the computational considerations. Consider using the spline base-learner functions for boosting the generalized additive model (GAM). In order to fit a multivariate spline model with respect to interactions, the number of knots in the spline grid will grow exponentially with the number of variables.

**Figure 3:**
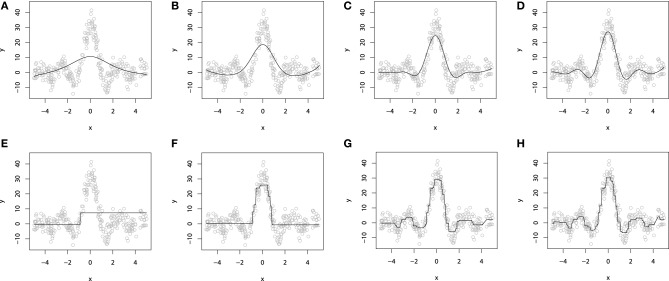
**P-Spline GBM model for different numbers of boosts: (A) *M* = 1; (B) *M* = 10; (C) *M* = 50; (D) *M* = 100**. Decision-tree based GBM model for different numbers of boosts: **(E)** *M* = 1; **(F)** *M* = 10; **(G)** *M* = 50; **(H)** *M* = 100.

To illustrate the additive GBM model with additive base-learners, we again refer to the artificial dataset, simulated from sinc(*x*) function. For demonstrative purposes, we will omit the impulsive noise component and choose the *L*_2_ loss and use the smooth spline base-learner functions. To provide a better intuition into the process of fitting a smooth additive boosting models, the resulting fits are evaluated for different number of boosting iterations *M*. The resulting demonstration is presented on Figures 3A–D.

When *M* = 1, we obtain a single penalized spline model, partially fitting the central part of the wave function. When we increase the number of iterations *M*, the accuracy of the fitted model grows gradually until the function is fitted considerably well. More details on the properties of GLM and GAM boosting models can be found in Buhlmann (2006) and Schmid and Hothorn (2007).

#### 3.2.2. Decision tree base-learners

A computationally-feasible way of capturing interactions between variables in GBM models is based on using the decision tree models. Although interactions between several explanatory variables would remove the interpretability property of additive models, this can not be considered a significant drawback as there are still several tools for tree-based GBM interpretation.

The idea behind a decision tree is to partition the space of input variables into homogenous rectangle areas by a tree-based rule system. Each tree split corresponds to an if-then rule over some input variable. This structure of a decision tree naturally encodes and models the interactions between predictor variables. These trees are commonly parameterized with the number of splits, or equivalently, the *interaction depth*. It is also possible to have one of the variables be split in a particular several times.

A special case of a decision tree with only one split (i.e., a tree with two terminal nodes) is called a tree stump. Therefore, if one wants to fit an additive model with tree base-learners, it is possible to do this using the tree stumps. In many practical applications small trees and tree-stumps provide considerably accurate results (Wenxin, 2002). Moreover, there is much evidence that even complex models with rich tree structure (*interaction depth* > 20) provide almost no benefit over compact trees (*interaction depth* ≈ 5).

One important property of the decision trees is that by design, a single decision tree always extrapolates the function with the constant value. An implication of this is that even a simple function like a straight line with a non-zero angle can not be approximated correctly with a single decision tree.

To demonstrate a GBM designed with the decision tree base-learners, we will use the same sinc(*x*) dataset as we used to illustrate the additive models. For this experiment, we also used the *L*_2_ loss. As the dimension of the explanatory variables is equal to one, we chose to use the tree-stumps. The resulting fitted models are shown on Figures 3E–H).

To demonstrate the progress of the fitting procedure, the number of iterations *M* was varied from 1 to 500. The similar behavior of consecutive improvements in the fit accuracy, when the number of iterations *M* increases, is apparent on this chart.

To conclude this section we must note that although there is a wide variability of possible design options, in most practical tasks one doesn't have to exhaustively try every possible combination of them. The choice of the loss function is often a matter of a particular task, whether to make the model more robust or not. Therefore, we advise the practitioners to first try fitting their models with classical loss functions, i.e., *L*_2_ loss for regression and Bernoulli loss for classification. As for the base-learner model, we would recommend to first try using tree stumps or low-interaction trees, because they usually perform reasonably well on many real-world datasets.

## 4. Regularization

The most important concern about building a machine-learning model from data is the resulting model's generalization capabilities. If the learning algorithm is not applied properly, the model can easily overfit the data. This means that it will predict the training data itself rather than the functional dependence between input and response variables. These concerns are obviously the same for GBMs.

**Figure 4:**
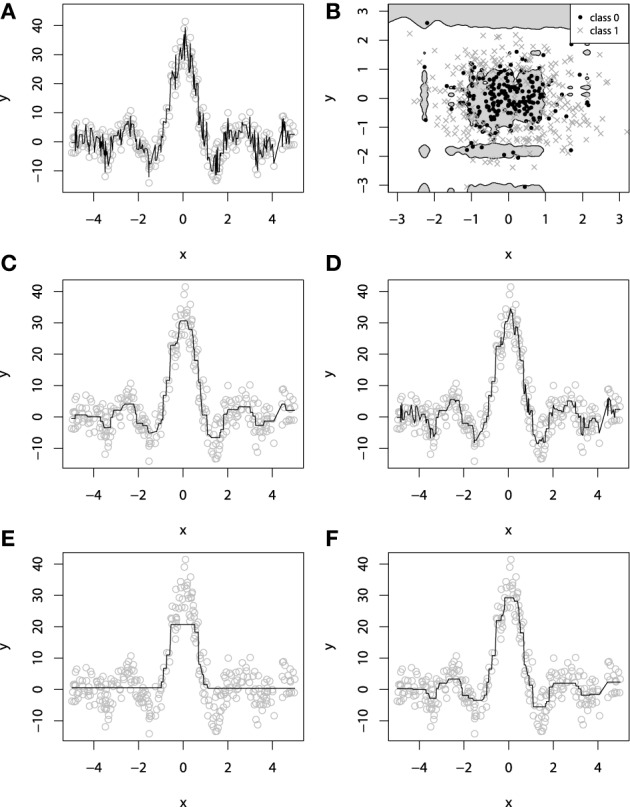
**Examples of overfitting in GBMs on: **(A)** regression task; **(B)** classification task**. Demonstration of fitting a decision-tree GBM to a noisy sinc(*x*) data: **(C)** *M* = 100, λ = 1; **(D)** *M* = 1000, λ = 1; **(E)** *M* = 100, λ = 0.1; **(F)** *M* = 1000, λ = 0.1.

It is easy to imagine a situation where new base-learners are added to the ensemble until the data is completely overfitted. Overfitting a GBM is possible with different types of base-learners with very different loss-functions. On Figures 4A,B, we illustrate overfitting for both regression and classification tasks.

To decrease the overfitting effects in GBMs, a number of different approaches were introduced. They help to constrain the fitting procedure and thus balance the predictive performance of the resulting model (Sutton, 2005; Zhang and Yu, 2005; Zou and Hastie, 2005). In this section, we will describe the most efficient regularization techniques that are most frequently used in GBMs.

### 4.1. Subsampling

The simplest of the regularization procedures introduced for GBMs is subsampling. The subsampling procedure has shown to improve the generalization properties of the model, at the same time reducing the required computation efforts (Sutton, 2005).

The idea behind this method is to introduce some randomness into the fitting procedure. At each learning iteration only a random part of the training data is used to fit a consecutive base-learner. The training data is typically sampled without replacement, however, replacement sampling, just as it is done in bootstrapping, is yet another possible design choice.

The subsampling procedure requires a parameter called the “bag fraction.” Bag fraction is a positive value not greater than one, which specifies the ratio of the data to be used at each iteration. For example, *bag* = 0.1 corresponds to sampling and using only 10% of the data at each iteration. Another useful property of the subsampling is that it naturally adapts the GBM learning procedures to large datasets when there is no reason to use all the potentially enormous amounts of data at once.

When the amount of data, measured by the number of data points *N* is not of practical concern, setting the default value *bag* = 0.5 gives a reasonable result for many practical tasks. If an optimal bag fraction is of interest, one can simply estimate it by comparing predictive performance under different parameter values.

However, one should also consider the effect of reducing the sample size on the model estimates. If the number of points becomes too low, one might receive a poorly fit model due to the lack of degrees of freedom. Therefore, some basic sanity-check analysis is essential before reducing the sample size.

It is also important to note the “big data” argument, as a consequence of the sample size reduction. In general, the more data there is available for the fitting a base-learner, the more accurate will the estimate be, if sufficient data was used. Therefore, when there are large amounts of data, one may consider a trade-off between the number of points, used for fitting each of the base-learners and the accuracy improvement, achieved by each of the base-learners.

One can easily arrive at a situation, when it is more efficient to have a larger number of base-learners, learnt with the lower *bag* rate. This means that the GBM ensemble will reach the desired accuracy with a larger number of base-learners and lower *bag* than the one with smaller amount of more carefully fitted base-learners with larger *bag*.

### 4.2. Shrinkage

The classic approach to controlling the model complexity is the introduction of the regularization through shrinkage. Shrinkage is commonly used in ridge regression where it literally shrinks regression coefficients to zero and, thus, reduces the impact of potentially unstable regression coefficients.

In the context of GBMs, shrinkage is used for reducing, or shrinking, the impact of each additional fitted base-learner. It reduces the size of incremental steps and thus penalizes the importance of each consecutive iteration. The intuition behind this technique is that it is better to improve a model by taking many small steps than by taking fewer large steps. If one of the boosting iterations turns out to be erroneous, its negative impact can be easily corrected in subsequent steps.

The simplest form of regularization through shrinkage is the direct proportional shrinkage (Friedman, 2001; Hothorn et al., 2010). In this case the effect of shrinkage is directly defined as the parameter λ ∈ (0, 1]. The regularization is applied to the final step in the gradient boosting algorithm:

(22) $${\hat{f}}_{t}\leftarrow {\hat{f}}_{t-1}+\lambda \rho_{t}h(x,\theta_{t})$$

It is a common pattern that the smaller parameter λ and therefore, the lower the shrinked boosted increments are, the better generalization is achieved. But, the cost of improving the generalization properties is the convergence speed. Choosing a stronger value of λ will increase the number of iterations *M*, required for convergence to a similar empirical loss minimum. For example, a decrease in λ by a factor of 10 implies an increase in the number of iterations *M* by a similar factor, slightly higher than 10.

An example of exploiting the shrinkage regularization is illustrated on Figures 4C–F. For this demonstration we used the *L*_2_ loss and the decision-tree base-learners. We didn't separate the dataset into training and validation set, because we wanted to show the geometric effects of shrinkage.

From Figure 4 we can deduce some interesting patterns. First of all, decreasing the shrinkage parameter requires more iterations to achieve the accuracy, compared to the non-regularized learning. Besides, we can see that using shrinkage results in capturing more details as it relies on a larger amount of boosts and thus, provides more continuity. This is especially important for decision-trees because, as previously discussed, they are very limited to capturing details by design.

Exploiting shrinkage in learning allows the decision-tree GBMs to capture more continuity in the modeled effects. The same effect of smoothing the decision-tree ensemble would also hold true for higher dimensional data, and that's why authors claim that it is desirable to train GBMs with infinitesimal step-sizes (Friedman, 2001; Buhlmann, 2006).

Analyzing Figure 4, one can note the effect of overfitting on the Figure 4D. This naturally leads us to the question of how does the shrinkage affect overfitting, or in the case of GBM, how does it affect the dependence between the learning error and the number of iterations.

**Figure 5:**
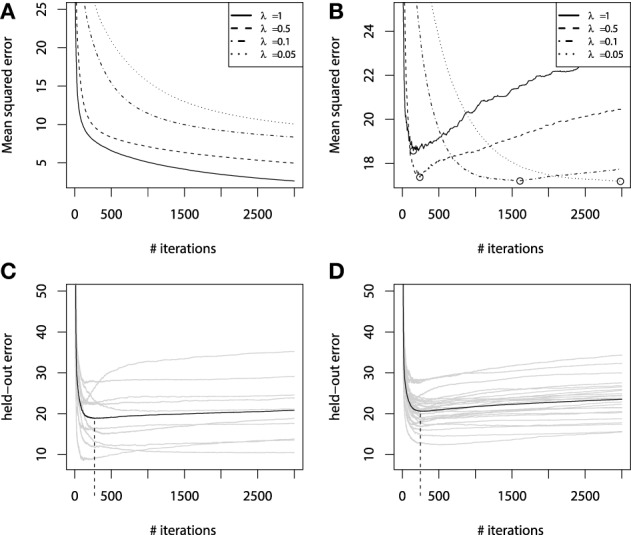
**Error curves for GBM fitting on sinc(*x*) data: **(A)** training set error; **(B)** validation set error**. Error curves for learning simulations and number of base-learners *M* estimation: **(C)** error curves for cross-validation; **(D)** error curves for bootstrap estimates.

To investigate this question in more detail, let us now consider the fitting experiment with both training and validation sets. Of the 300 initial points, we use randomly resampled 200 of them for training, and another 100 for validation. All the other experiment parameters remain unchanged. The learning error curves for GBMs with different λ parameters are presented on Figure 5.

From Figure 5A we can see that the training set error is substantially falling, but the speed of this improvement heavily depends on the shrinkage parameter λ. A much more important effect from a practical point of view is the validation set error behavior, which is shown on Figure 5B. The validation-error hyperparameter *M*, corresponding to the error minima of each of the models, is highlighted with circles. We can see that increasing the shrinkage leads to both finding a better hyperparameter *M* minima and to improving the generalization of the model. The latter corresponds to the fact that shrinked models have a flatter plateau beyond their error minimas, and it takes them many more iterations to initiate overfitting. Yet, it also means that these models will naturally take longer to learn.

### 4.3. Early stopping

Using regularization techniques described above, one can significantly improve the generalization properties of a GBM model. However, given a shrinkage parameter λ, the optimal number of iterations *M*_opt_, in the sense of the validation set performance, can be different from the initially pre-specified one *M*. We have illustrated this phenomenon on the Figure 5.

Once important practical consideration that can be derived from Figure 5 is that one can greatly benefit from early stopping (Zhang and Yu, 2005). This means that if the ensemble was trimmed by the number of trees, corresponding to the validation set minima on the error curve, the overfitting would be circumvented at the minimal accuracy expense. Another observation is that the optimal number of boosts, at which the early stopping is considered, varies with respect to the shrinkage parameter λ. Therefore, a trade-off between the number of boosts and λ should be considered.

In practice one typically chooses the shrinkage parameter λ beforehand and varies the number of iterations *M* with respect to the chosen shrinkage. One possible approach to choosing the number of iterations *M* would be to use an information criterion like Akaike's AIC or some sort of minimum description length criteria. However, they have been shown to overshoot the true number of iterations (Hastie, 2007) and thus are not recommended for practical usage.

The most frequently used approach to deal with this trade-off relies on the cross-validation procedure. Cross-validation provides means for testing the model on withheld portions of data, while still using all of the data at the processing stages.

First, the shrinkage parameter λ, the maximum number of iterations *M*_max_ and the cross-validation parameter *k*, corresponding to the number of validation folds, are specified. The data is then partitioned into *k* disjoint non-overlapping subsets. Afterwards, for each of the *k* subsets of the data, one of them is set aside as the validation set and the others are used for fitting a GBM model. The fitted GBM is then tested on the validation set to produce the held-out estimates of the predictive performance. At last, the validation performance is aggregated from each of the folds, for example, by averaging the validation set performances. This aggregated measure serves as estimate of model generalization on the validation set.

One may note that besides cross-validation one can use a similar procedure to test the model on bootstrap samples (Hofner et al., 2012). Bootstrap is essentially useful for parameter estimation when the training dataset is considerably small. In bootstrapping, we choose the number of bootstrap samples *B* similarly to the number of folds *k* in cross-validation. Afterwards, at each iteration we randomly sample with replacement the original data, which leads to approximately 63% of the unique original data entries in each sample. This means that if we had a sample of {1,2,3}, the resulting bootstrap samples can, for example, be {1,1,3} or {3,2,2}. The held-out estimates are evaluated on the left-out original data entries, the so-called “out of the bag” values. These values are then aggregated in the same fashion, as in the cross-validation.

The results of this procedure are illustrated in Figures 5C,D. For this experiment we used the same parameter setting as in all the other regularization experiments, with the same training and validation sets. For the hyperparameter specification we chose λ = 0.5, *M*_max_ = 3000, *k* = 10 folds for cross-validations and *B* = 25 for boostsrapping.

As we can see from the simulation plots, the average behavior of the held-out errors is rather similar. And from both of these plots we can deduce very similar estimates of the optimal number of iterations *M*. Namely, the cross-validation estimate is *M*^CV^_opt_ = 255, the bootstrap estimate is *M*^boot^_opt_ = 241, while the optimum of the validation set was *M*_opt_ = 245. It means that both methods provide us with considerably good estimates of the number of iterations.

## 5. Model interpretation

In practice, it can be of great utility to be able to interpret the resulting model. As we have previously discussed, additive GBM models can be trivially explained, as the additive components correspond to the marginal dependence plots by design. One only has to predict each additive component over a grid of values of the corresponding variable and plot it.

When one uses an ensemble of decision trees with high interaction depth, the same visualization approach is inapplicable. And despite the simplicity of a simple decision tree, when there are thousands of trees in the ensemble it becomes challenging to interpret such models. However, even decision tree GBMs can be interpreted with the appropriate tools.

Several tools have been designed to alleviate interpretation problems in decision-tree based GBMs. Therefore, even high interaction-based GBMs should not be considered completely black boxes, as the resulting models can provide important insights into the captured dependencies. In this section we describe the most common tools for GBM interpretation.

### 5.1. Relative variable influence

A common practical task is to identify the variable importance. To perform feature selection in decision-tree ensembles the main modeled effects are not separated from the effects caused by interactions. Therefore, one cannot strictly analyze the captured effects in a similar fashion to the regression coefficients. For this purpose, the variable influence for the decision tree ensembles, based on the decision trees influences(Breiman et al., 1983), was proposed (Friedman, 2001).

If we consider a likelihood framework of GBMs, and for simplicity assume the *L*_2_ loss, it follows that the increase in log likelihood is proportional to the increase in sums of squares explained by the model. Each split on a variable in a decision tree increases the log likelihood of the whole ensemble and the sum of log likelihood increases across all trees.

Let us define the influence of the variable *j* in a single tree *T*. Consider that the tree has *L* splits, therefore we are looking for all the non-terminal nodes from the root to the *L* − 1 level of the tree. This gives rise to the definition of the variable influence:

(23) $${\text{Influence}}_{j}(T)=\sum_{i=1}^{L-1}I_{i}^{2}1(S_{i}=j)$$

This measure is based on the number of times a variable is selected for splitting, i.e., current splitting variable *S*_*i*_ is the same as the queried variable *j*. The measure also captures weights of the influence with the empirical squared improvement *I*^2^_*i*_, assigned to the model as a result of this split. To obtain the overall influence of the variable *j* in the ensemble, this influence should be averaged over all trees.

(24) $${\text{Influence}}_{j}=\frac{1}{M}\sum_{i=1}^{M}{\text{Influence}}_{j}(T_{i})$$

The influences are further standardized so that they add up to 100%. Influences do not provide any explanations about how the variable actually affects the response. The resulting influences can then be used for both forward and backward feature selection procedures.

### 5.2. Partial dependence plots

Visualization is one of the most comprehensive ways of interpretation. We have already stated that additive GBMs can be plotted fairly easily. In decision-tree GBMs similar model representation can be achieved with partial dependence plots. Partial dependence implies the demonstration of the effect of a variable on the modeled response after marginalizing out all other explanatory variables.

Although the correct way of obtaining the marginal plots would be to numerically integrate out other variables over a suitable grid of values, it can be very computationally consuming in practice. An easier approach is therefore commonly used, when the marginalized variables get fixed with a constant value, equal to their sample mean.

These graphs might not be a perfect representation of the captured effects, especially if the variable interactions significantly impact the resulting model. However, partial dependence plots can provide a useful basis for interpretation that has been noted practical in different applications (De'ath, 2007; Hutchinson et al., 2011; Pittman and Brown, 2011).

The same idea with visualization can be applied to couples of variables, therefore allowing one to inspect and analyze the most important interactions. To identify the interactions of interest, one might first use the relative variable influence and then produce pairwise dependence plots.

We shall illustrate the described interpretations options in the following section on several real world application examples.

## 6. Applications

In the previous sections we have discussed various aspects of the GBM design on synthetic and toy data examples. In this section we will provide explicit walkthroughs of applying GBMs to several real-world applications. All of the considered models were evaluated in R programming language with *gbm* and *mboost* packages. Although ensemble models are considered more resource-consuming than their competitor methods, a single PC will be enough for most applications that do not deal with the Big Data. For all of the described applications in this section a single Windows PC with Intel Core i7-2670QM and 12GB of RAM was used.

### 6.1. EMG robotic ARM controller

In our first practical application we would consider building a regression model to map the EMG signals to the robotic hand controller, in a manner described in Vogel et al. (2011). The data was provided by the TUM Roboterhalle machine learning laboratory.

In this application, we will focus on walking through the whole GBM model application solution, where our main focus will be on investigating the properties of different base-learner models.

#### 6.1.1. Application description

In the original setting (Vogel et al., 2011), nine surface EMG electrodes, positioned on the hand, were used to record the muscular activity of the person performing different hand movements. These movements were then visually tracked to gather the actual spatial positions of the hand. Combined, this data was used to design a robotic arm control system. The machine learning task was intended to reconstruct the hand's position and orientation from the EMG channels and then to use it online as the robotic hand control.

In our application we would consider a slightly altered experiment setup than the originally described one (Vogel et al., 2011). In our case, we will have only eight EMG channels available for modeling. The 9th channel was omitted due to experiment design considerations.

To make the application walkthrough easier, we will focus our analysis on predicting only the first output variable, which corresponds to the first coordinate of the hand position. This is done to simplify the examples and focus on the GBM design specifics. Predicting other output variables is equivalent to building another GBM models for each of the variables. Yet the final accuracy results are provided for all the hand positions simultaneously.

#### 6.1.2. Data processing

To proceed with the analysis, data has to be properly processed. At first, the absolute values of the EMG channels are taken. Next, due to the fact that the sampling frequency of the EMG channels is 10 times higher than that of the camera-tracking, the absolute values of the EMG signals are chunked into 10-point intervals. Afterwards the maximum values over these intervals are used to form a new feature, which is of the same sampling frequency and sample length as the target output variables. At this point, the feature proxy is ready, and can already be fed into the GBM model.

The resulting pre-processed signals can then be used for further feature extraction. The simplest design choice is to smooth the signal with the moving average, however, one can consider mining more sophisticated features like the rolling standard deviations. In this application we will assume that the original positions of the hand can be efficiently reconstructed with the low-frequency components of the EMG signals only.

**Figure 6:**
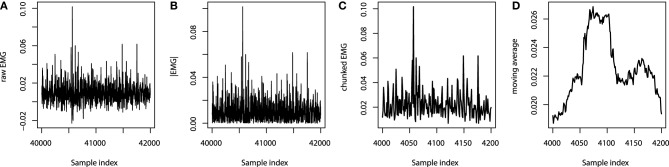
**EMG processing: (A) raw EMG data; (B) absolute EMG sensor values; (C) chunked EMG data; (D) moving average smoothed data**.

We chose to extract only the moving averages with the sliding window width chosen to be 50 points, which is equivalent to the last 500 ms of readings. The overview of the feature processing of one particular EMG channel is given on Figure 6. Note that the sets of indices on Figures 6A–D are different due to different sampling rates.

After the processing is finished, we arrive at eight signal features and 27,161 observations. Originally, the controller was first trained on all of the available data and then tested live, without knowing the correct labels, as the hand-tracking device was not available. In our case, we train the models on one half the available data and validate it on the other part. The train/test separation is organized sequentially: the first 100 points are used for training, the following 100 for validation, the next 100 points are used for training again and so on. As a consequence, the training set consists of 13,561 points and the test set consists of 13,600 points.

#### 6.1.3. GBM design

Our primary concern in this application is to model the conditional expectation of the target output variable. The model performance metric to be used will be the root mean squared error (RMSE), evaluated for each of the position variables *y*_*i*_, *i* = 1, 2, 3:

(25) $${\text{RMSE}}_{i}=\sqrt{\sum_{j=1}^{N}\frac{1}{N}{\left(y_{ij}-\hat{y_{ij}}\right)}^{2}}$$

The compiled, 3D error metric shall be defined as:

(26) $$\text{M3DE}=\sum_{i=1}^{N}\frac{1}{N}\sqrt{{\left(y_{1i}-\hat{y_{1i}}\right)}^{2}+{\left(y_{2i}-\hat{y_{2i}}\right)}^{2}+{\left(y_{3i}-\hat{y_{3i}}\right)}^{2}}$$

As the process of waving hands was very continuous, the target value distribution is not significantly affected by outliers and severe distribution-violating artifacts. Therefore, we will consider the conventional *L*_2_ loss for our purposes.

After we have specified the loss-function, we have to choose the base-learner model. As the primary objective of this application is to describe properties of different base-learner models on the real-world application, we will proceed with consecutive building four GBMs with the most frequently used base-learner models.

We shall proceed with boosting the additive GBM models, at first applying the linear base-learners and then the spline learners. Afterwards, the tree based base-learners will be applied to the same learning task in both additive and interaction-based forms. At last, we shall compare the models based on their performance accuracy on the held-out test set.

The next design choice is for the learning hyperparameters, specifically the number of boosts *M* and the regularization λ. As stated in the regularization section of the article, one can estimate the number of boosting iterations *M* with the help of either bootstrapping or cross-validation with respect to previously-chosen value of λ.

Since we didn't have any prior information, we set parameters λ = 0.01, *M*_max_ = 1000 and proceed with the bootstrap estimates of *M*. Setting λ = 0.01 is some sort of the default value. Using lower values of the regularization parameter will consider higher awareness of overfitting. For estimating the optimal number of iteration *M* we take *B* = 25.

#### 6.1.4. Model evaluation

Now we can proceed to the GBM model evaluation with the above mentioned design and hyperparameter settings. We remind the reader, that all of the aforementioned analysis is provided for building a regression model for the first positional variable only.

**Figure 7:**
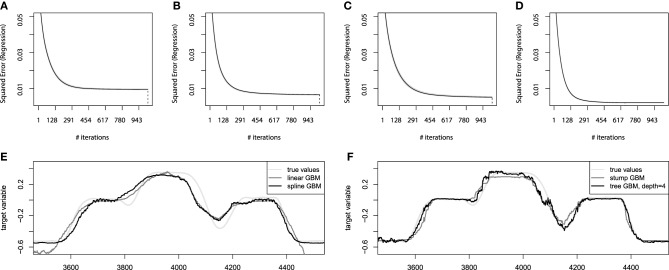
**Bootstrap estimates of *M* for the EMG robotic control data**. **(A)** Held-out error for linear GBMs; **(B)** held-out error for spline GBMs; **(C)** held-out error for stump-based GBMs; **(D)** held-out error for tree-based GBMs with interaction depth *d* = 4. **(E)** Sample prediction of the additive GBMs for the EMG robotic control data; **(F)** sample prediction of the tree-based GBMs for the EMG robotic control data.

At first, we shall consider the estimates of the optimal number of iterations for the additive GBM models. We shall infer these estimates from the out-of-bag estimates on the convergence plots of these models. The corresponding convergence plots with the bootstrap estimates of the number of iterations are presented on Figures 7A,B.

From these convergence plots several implications can be deduced. The first of them is the high compactness of the bootstrap estimates (gray lines). This means that the data is very concise and the chosen hyperparameter setup is generalizing the data well. Another implication is that the number of boosting iterations was chosen of the appropriate scale for both additive models. The linear model on Figure 7A could even have the number of iterations lowered twice without significant loss of accuracy. And the spline GBM model on Figure 7B can actually have the number of iterations increased, although this won't contribute to the performance dramatically as the result already behaves as if the model has converged.

After the additive models are learnt, we can validate them on the held-out test set. The linear GBM achieves the Mean 3D error = 0.136 performance on the test set, while the spline-based GBM reaches Mean 3D error = 0.105. This difference will become even more noticeable if we compare the obtained predictions of both models. The sample predictions are presented on Figure 7E. It is clear that both models approximate the function considerably well, although the linear one is less accurate due to its design simplicity.

The next step of the analysis is to apply the tree-based GBM models. We will follow the same hyperparameter settings, *M*_max_ = 1000, λ = 0.01, and *B* = 25. To make a better illustration of the importance of modeling the interactions, we will analyze two tree-based GBMs: the boosted stumps and boosting the trees with interaction depth of 4. The choice of the interaction depth is heuristic-based and could be analyzed in more detail, but we consider the chosen level of interactions suitable. The corresponding convergence plots for the tree-based GBMs are presented on Figures 7C,D.

From the convergence plots on Figures 7C,D we can make some new, model-specific implications. First of all, the stump-based GBM achieves nearly the same accuracy as the spline-based model. And the tree-based model with higher interaction depth is considerably more accurate than any of the GBM models built. Moreover, due to the increased model complexity, the convergence was achieved much faster, which means that the optimal number of iterations *M* for the tree-based GBM is approximately 650 instead of a 1000.

In terms of the resulting accuracy, the stump-based GBM reaches the Mean 3D error = 0.104 performance on the test set, while the higher interaction tree-based GBM reaches Mean 3D error = 0.081. This difference becomes more appealing if we compare the resulting prediction plots of both models like we did previously for additive models. The sample predictions are presented on Figure 7F.

We can see that the stump-based GBM not only achieves nearly the same accuracy and convergence rates, but also predicts values very similar to the ones predicted by the spline-based GBM. And the GBM with trees of higher interaction depth achieve a visually noticeable better prediction accuracy. These predictions are still not perfect, as the capability of designing a perfect mapping from the available features might be not possible at all. However, the resulting model achieves reasonably high accuracy.

So far we have been investigating the accuracy of different GBM model designs. To evidence the usefulness of this method, we will apply other popular machine learning techniques and compare their obtained performances. The chosen methods are the Linear Regression, the Support Vector Machine(SVM) with radial kernel and the Random Forest (RF). The optimal hyperparameters for the SVM and RF models were chosen by the fivefold cross-validation applied to the grid-search. The algorithm accuracy comparisons are given in Table 1.

**Table 1:** **Machine learning algorithm accuracy**.

**Method**	**RMSE_1_**	**RMSE_2_**	**RMSE_3_**	**M3DE**
GBM, linear	0.100	0.087	0.095	0.136
GBM, spline	0.081	0.063	0.084	0.105
GBM, stumps	0.079	0.063	0.085	0.104
GBM, trees, *d* = 4	0.063	0.054	0.066	0.081
Linear regression	0.100	0.087	0.095	0.136
Support vector machine	0.076	0.069	0.084	0.100
Random forests	0.062	0.054	0.067	0.081

We note that both the tree-based GBMs and the RF reach similar high accuracy. However, one should be aware that the RF builds trees as deep as needed, therefore modeling much more accurate and complex interaction structure. Yet, increasing the GBM tree-depth to *d* = 8 didn't give any increase in accuracy. So we can say that both ensemble techniques are almost equally accurate on this data, maybe in some sense complementary, as these methods behave slightly better than one another on different target variables. Therefore, the GBMs achieve the highest possible accuracy on the mined features, sharing its first place with RF.

#### 6.1.5. Model interpretation

**Figure 8:**
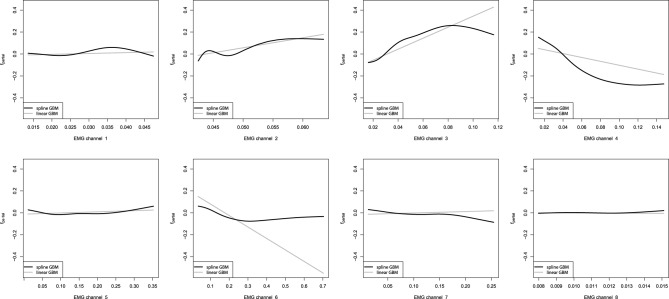
**Partial dependence plots of additive GBMs for the EMG robotic control data**. The gray line corresponds to the linear GBM; the black line corresponds to the spline-based GBM.

After we have built the GBM models, we want to investigate the captured dependencies. At first we will analyze the partial dependence plots of the built GBMs. We shall start with the additive GBM models, as the detailed analysis of the resulting models obtained is trivial. These plots will be especially descriptive due to the low dimensionality of the learning problem. The partial dependence plots for the additive GBMs are presented on Figure 8.

From the obtained partial dependence plots, we can see that both linear and spline models are considerably similar in the effects they capture. Another interesting property is that the linear GBM resulted in a sparser model than the one, which could be achieved by performing simple LSE linear regression estimation. Namely, the eighth EMG channel turned out to be omitted by the sequential boosted learning, assigning it the zero coefficient and thus discarding it from the resulting formula. However, the standard Linear Regression model also assigned the eighth channel a very low coefficient, which had an insignificant *t*-statistic, thus, meaning that it would have been dropped off by the conventional analysis too.

**Figure 9:**
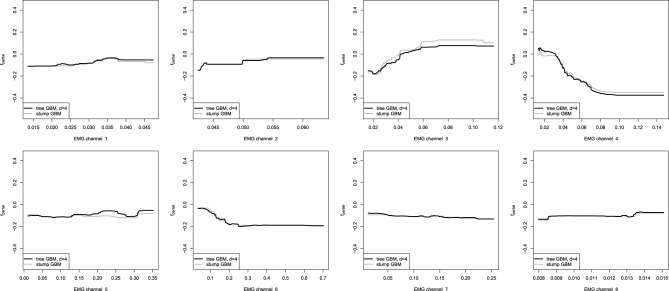
**Partial dependence plots for tree-based GBMs on the EMG robotic control data**. The gray line corresponds to the stump-based GBM; the black line corresponds to the tree-based GBM with interaction depth *d* = 4.

Our next step is to analyze the partial dependence plots of the tree-based GBMs. We must note that the partial dependence plots for the non-stump GBM was obtained by using the average values of the marginalized parameters due to the non-trivial interaction model. The resulting partial dependence plots of the tree-based GBMs are shown on Figure 9.

One can easily see that the partial dependence plots of both the stump and non-stump GBMs are very similar, even though they were obtained by slightly different procedures. Moreover, partial dependence plots of the spline-based GBM on Figure 8 provide very similar results as compared to the obtained tree-based plots. This can be explained by the fact that we have captured very similar patterns and dependencies in the data.

**Figure 10:**
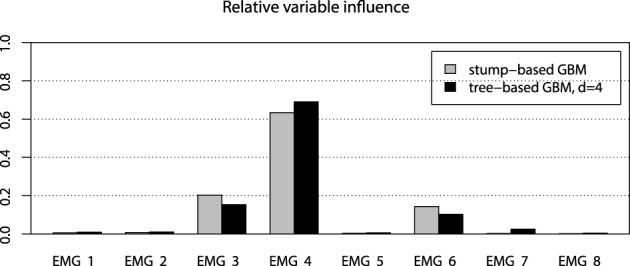
**Relative variable influence for tree-based GBMs on the EMG robotic control data**.

However, the non-stump tree GBM achieved a higher accuracy than both the stump-based and the spline-based models, having almost similar marginal partial dependencies. We are interested in visualizing the interactions that can be of high interest for the practitioner in order to analyze the resulting model. As we have noted in the previous section, the tree-based ensemble methods have the special relative variable influence statistic to capture the relative variable importance in the presence of a large number of random interaction effects. The mined relative variable influence statistic from both tree-based GBMs is given on Figure 10.

From the variable influence two important facts can be outlined. First of all, the resulting model mostly depends on three EMG sensors only, which brings in considerations for potential dimension reduction, namely the third, fourth and sixth channels. Another point is that the effect of the captured interactions must increase the relative influence of a variable, compared to its influence in the stump-based GBM. This positive difference is most noticeable for the fourth and seventh channels. To investigate the pairwise interactions, we therefore chose the pairs of (3,4) channels and (4,7) for visualization.

**Figure 11:**
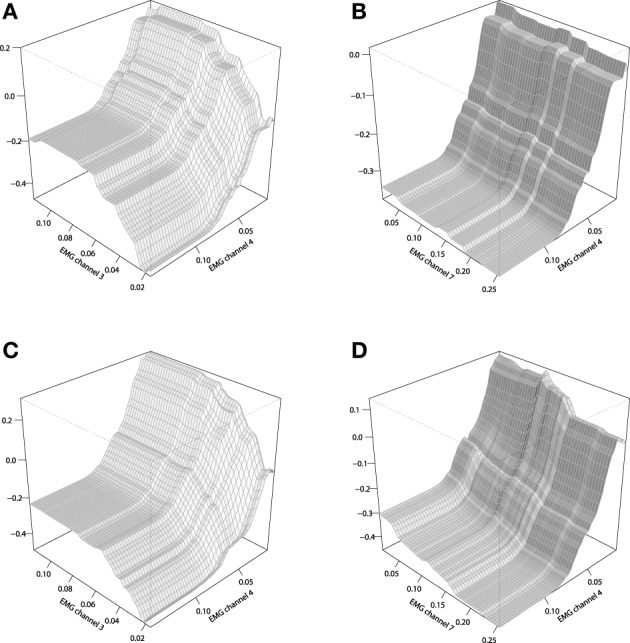
**Interaction plots of the tree-based GBMs on the EMG robotic control data**. **(A)** stump-based GBM, channels 3 and 4; **(B)** stump-based GBM, channels 4 and 7; **(C)** tree-based GBM (*d* = 4), channels 3 and 4; **(D)** tree-based GBM (*d* = 4), channels 4 and 7.

To analyze the chosen interaction effects, we shall consider plotting the 2-dimensional interaction plots. These plots are generated by averaging out all the input variables except for the ones that were chosen for building a chart. The corresponding interaction plots are given on Figure 11, built in comparison with the stump-based trivial interaction structure.

One can see that the difference between that two interaction plots look considerable, but the second pair of channels (4,7) provides a more significant difference between the two GBM models of Figures 11B,D. These plots could be used by the EMG experts to get a better understanding of the resulting control model and its underlying effects.

#### 6.1.6. Application conclusion

In this application we have shown how to successfully build GBM models with different base-learner models and how do the results have to look like. The resulting models provided good performance results in the sense of predictive accuracy, as compared to the commonly used machine learning techniques. Afterwards, we have extensively shown how to interpret the obtained GBM models with respect to the base-learner model, used in designing a particular GBM.

### 6.2. EMG physical action classification

In our previous practical example we built a regression model for robotic control based on readings from EMG sensors. For our next example we will also consider exploitation of the EMG readings, but this time they will be used for monitoring and classifying human physical activities. The dataset for EMG-based physical activity classification was obtained from the UCI Machine Learning Repository (Bache and Lichman, 2013). We will use only the set of measurements of the first EMG experiment participant, thus omitting the data from the three other subjects.

In this application, we will focus on all the stages of building a GBM model solution for the classification example.

#### 6.2.1. Application description

The learning task for this dataset is to build a physical activity classifier, based on the EMG sensor readings. There are 20 classes, each corresponding to a particular type of activity. These classes are then grouped into two metaclasses of activities: normal and aggressive ones. The list of classes is given on Table 2.

**Table 2:** **Physical action classes**.

Normal	Bowing, clapping, handshaking, hugging, jumping, running, seating, standing, walking, waving
Aggressive	Elbowing, frontkicking, hamering, headering, kneeing, pulling, punching, pushing, sidekicking, slapping

We will consider learning the task of correctly classifying the EMG activity vectors into each of the 20 given classes.

#### 6.2.2. Data processing

The number of EMG channels in this dataset is also equal to 8. The data processing is carried out in exactly the same way as the procedure, previously applied to the EMG robotic controller data. Specifically, at first the absolute value of the EMG channels is taken, afterwards it is chunked into 10-point intervals with their maximum values, and at last the 10-point moving average filter is applied.

After the data processing is finished, we arrive at a dataset of 18,691 points. We follow the same consideration of training and test set separation, so the training set consists of 9300 points and the test set of 9391 points.

#### 6.2.3. GBM design

In this application we are mostly concerned with the class-wise accuracies for each of the classes *C*_*i*_, *i* = 1, …, 20. If we consider the output of the GBM as the class-label, equivalent to the class with the highest GBM output value, the class-wise accuracy is formulated as follows:

(27) $$E_{C_{i}}=\frac{1}{N}\sum_{i=1}^{N}\delta (\hat{f}(x_{i}),y_{i})$$

The $\delta (\hat{f}(x_{i}),\;y_{i})$ is the Kronecker's delta function, which equals to 1, when the values coincide, and 0 otherwise. As there are 20 classes considered, using more sophisticated accuracy metrics is complicated, thus, for the first classification task we will use the average class-wise accuracy *E*_average_:

(28) $$E_{\text{average}}=\frac{1}{20}\sum_{i=1}^{20}E_{C_{i}}$$

As the data consists of 20 classes, we will follow the standard approach of learning a multi-class model in a “one vs. all” fashion. This means that we will consecutively build 20 one-class GBMs, learned to classify only one distinct class. The “one vs. all” approach is common to other classification algorithms like SVMs (Rifkin and Klautau, 2004). Just as in the previous EMG example, we will analyze one particular GBM model for the first class in more detail, however, we will also provide the resulting obtained accuracy for the whole 20-class problem.

When building a binary, 2-class classifier with the GBM models, it is desired to have both classes to share some reasonable portions of the data, like 50% of the points per class. However, even though the distribution of the class labels in the dataset is nearly uniform (classes are represented with equal frequencies), “one vs. all” classifiers will have this balance dramatically different. Approximately 5% of the points will be relevant to the desired class in each of the 20 models. To compensate for this effect, classes receive an additional weight vector, assigning weights of *w*_*fp*_ = 20 to the false positives error and *w*_*fn*_ = 1 to the false negatives. The weights are then simply multiplied by the classification error at each iteration of the learning process.

The GBM design choices for this application will be similar to the ones, used in our previous EMG application. The most accurate results were previously achieved on the EMG data with the GBMs, taking interaction effects into account. In this application we will concentrate on using tree-based GBMs as the base-learner models, with the initial interaction depth set to *d* = 4. The choice of the loss function doesn't require any specific customization, therefore we will use the Bernoulli loss for this application.

#### 6.2.4. Model evaluation

**Figure 12:**
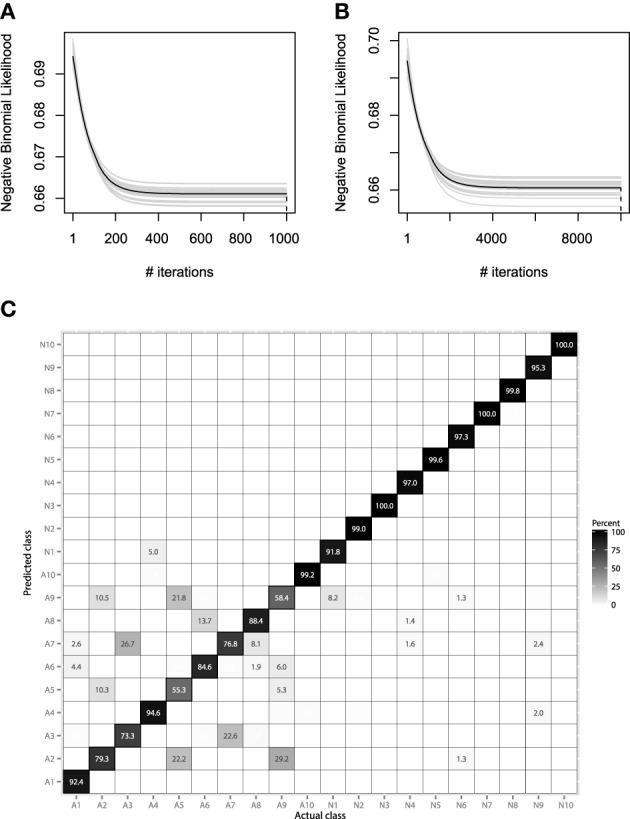
**(A)** Bootstrap estimates of *M* for the EMG classification data, held-out error with λ = 0.01. **(B)** Bootstrap estimates of *M* for the EMG classification data, held-out error with λ = 0.001. **(C)** Confusion matrix for the EMG activity classification test set.

We will consider using the same hyperparameter values, as in the previous EMG example. Namely, the hyperparameter choice is λ = 0.01, *M*_max_ = 1000, and *B* = 25. More accurate learning parameters, λ = 0.001, *M*_max_ = 10,000, will also be considered, as the learning process will be carried out much slower and thus, will hopefully lead to more accurate results. The resulting convergence plots are given on Figures 12A,B.

Both convergence plots look very similar and motivate to decrease the number of boosting iterations by half. If we evaluate both models on the test data, we will receive nearly the same classification accuracy of 89.1%. This indicates that there is no need to perform overly accurate learning and that the standard guess of λ = 0.01 worked for this data well. The resulting confusion matrix for the 20-class problem is presented on Figure 12C.

The resulting confusion matrix shows that our method has achieved reasonably-high accuracy on this problem. One can note that the two meta-classes of aggressive and normal actions are very well separated, having a correct classification rate of 98.8%. If the metaclass binary classification problem was considered, one could achieve even higher accuracy, because class-wise specific details make the models more specific and thus, less generalizing.

It is also noticeable that the method fails at correctly predicting only the aggressive actions. Specifically, the highest misclassification rate is between the 2nd, 5th, and 9th classes. Namely, these classes correspond to the activities of frontkicking, kneeing and sidekicking. This might indicate that these classes of activities are naturally very hard to distinguish from the EMG measurements only. To finish the evaluation of the EMG classification, let us compare the performance of the obtained GBM model with other machine learning algorithms. We will validate the GBM performance with Logistic Regression (LR), SVM and RF. The optimal parameters for the non-linear methods were once again chosen by the fivefold cross-validation applied to the grid-search. The algorithm accuracy comparisons are given in Table 3.

**Table 3:** **Machine learning algorithm accuracy**.

**Method**	***E*_average_ (%)**
Logistic regression	84.7
Support vector machine	86.6
Random forest	84.8
GBM, trees, *d* = 4	89.1

#### 6.2.5. Model interpretation

**Figure 13:**
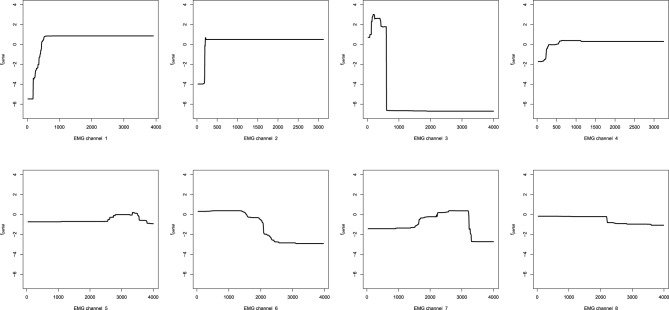
**Partial dependence plots for the EMG activity classifier based on the decision-tree GBM**.

In a classification task one can still investigate any of the previously defined visualization tools like partial dependence plots. For each of the consecutive 20 classifiers, values above or below zero would correspond to the contribution of labeling the queried point to the marginal classes −1, 1, i.e., “not in the class” and “belonging to the class,” respectively. The resulting partial dependence plots for the first class (Bowing) are given on Figure 13.

**Figure 14:**
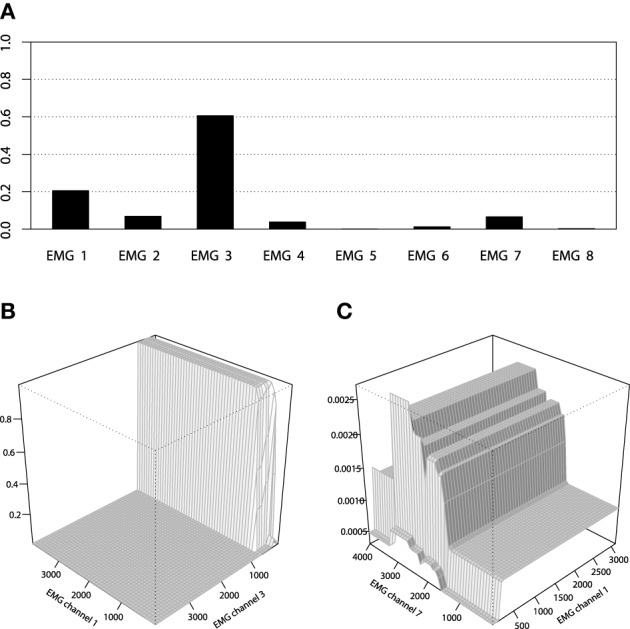
**(A)** Relative variable influence of the EMG activity classifier for Bowing class. **(B)** Interaction plot of the EMG activity classifier for Bowing class, channels 1 and 3. **(C)** Interaction plot of the EMG activity classifier for Bowing class, channels 1 and 7.

We can also apply the same inference tools to further analyze the resulting GBM model. For example, the relative variable influence of the obtained GBM model is given on Figure 14A and the 3D interaction plots are given on Figures 14B,C.

#### 6.2.6. Application conclusion

In this application we have shown how to apply the GBM models to the classification tasks, having the example take into account multiple classes. This time the GBMs, applied to the mined signal features, outperformed other methods considered as benchmarks in terms of accuracy. Yet it is worth noting that even the linear classifier worked reasonably well on this data. One can also consider mining more sophisticated features from the EMG channels, however, with even the simplest moving averages one can achieve high classification performance.

### 6.3. Text classification

One of the important properties of GBMs that we have previously mentioned is the possibility of building sparse models. This property can be desirable in a number of practical cases, for example when the predictor data comes from a very high dimensional distribution whilst containing very little, sparsely distributed information.

In this application we will focus on the specific GBM design, when the sparse model or the data is considered. A common example of such data is the so-called document-term matrices and similar data structures. The rows of a document term matrix correspond to a particular document and columns reflect the frequency of a particular word occurrence in this document. As the number of words is considerably high, many of them seldom appear in the set of the documents analyzed, thus showing zero frequency in most of the documents.

#### 6.3.1. Application description

We will consider the analysis of the GBM model performance on the CNAE-9 dataset (Bache and Lichman, 2013). This dataset was generated with the intent to automatically classify Brazilian companies based on their text descriptions into 9 classes, according to their economic activities. The data consists of 1080 rows, corresponding to documents, and has 856 columns, representing frequencies of particular words. A notable property of the data is that it is very sparse: 99.22% of the resulting matrix is filled with zeros.

#### 6.3.2. Data processing

The original data collection and processing are of lesser importance in this application, more details on these questions can be found in Ciarelli and Oliveira (2009). Here we would just apply the GBMs to the available dataset without any manipulations on its features, or any external expert-driven knowledge involved.

Due to the sparsity of the data, the previous approaches to solving this classification problem relied on different dimension reduction techniques (Ciarelli and Oliveira, 2009; Ciarelli et al., 2010). To make the processing even more simplified, we will consider building a sparse GBM model “off the shelf” by design. Just as we had to build a 20-class model in the EMG classification case study, we would be following the same strategy with this dataset. Specifically, we will be building nine GBM models for each class in the similar “one vs. all” fashion with each model weighted the same way as previously, with false positive weights *w*_*fn*_ = 9.

For the purposes of results comparison, we will use the common train and test set conventions as in the previous works that touched upon this dataset (Ciarelli and Oliveira, 2009; Ciarelli et al., 2010), taking the first 900 points for training and the remaining 180 points for testing the model. The final accuracy and the corresponding confusion matrix will therefore be assessed on the test set points.

#### 6.3.3. GBM design

In this application, due to having much more than two classes, we will once again consider the simple average accuracy *E*_average_ as the model evaluation criteria:

(29) $$E_{C_{i}}=\frac{1}{9}\sum_{i=1}^{9}E_{C_{i}}$$

Since we didn't have any prior information, we set parameters λ = 0.01, *M*_max_ = 1000 and proceed with the bootstrap estimates of *M*. Setting λ = 0.01 is some sort of the default value. Using lower values of the regularization parameter will consider higher awareness of overfitting. For estimating the optimal number of iteration *M* we take *B* = 25.

#### 6.3.4. Model evaluation

To build a GBM one has to choose the type of base-learners and the loss-function to optimize, plus several hyperparameters. As there is no specific need to modify the loss function, we will once again choose the Bernoulli loss. But the base-learner choice is significantly motivated by the data geometry. There is no need to introduce the smooth terms because the data is sparse and rarely contains values different from zero and one. Moreover, the choice of non-stump decision trees, (i.e., trees with non-trivial interactions) might bring the exceeding complexity into the model. It will result in the unstable fit, which will be prone to overfitting due to excessive leaves, corresponding to 0-levels of different variables. As a consequence, GLMs and tree-stumps would behave similarly due to the specifics of the described data distribution. Therefore we will consider only the GLM base-learner model.

After we have chosen the loss function and the type of base-learners, we have to specify the learning hyperparameters *M* and λ. In the particular setting, we want to add function increments as small and accurate as possible due to the high awareness of overfitting the data. The initial setup of λ = 0.01, *M*_max_ = 100,000 and the common *B* = 25 are a good startup for this experiment. It is worth noting that using subsampling has to be done very carefully, as one can easily arrive at completely degenerate variables with all zero-values, once again due to the sparsity of the data. In its turn, in this particular setting using cross-validation is less desirable than bootstrapping, however, both of these methods can lead to this problem.

**Figure 15:**
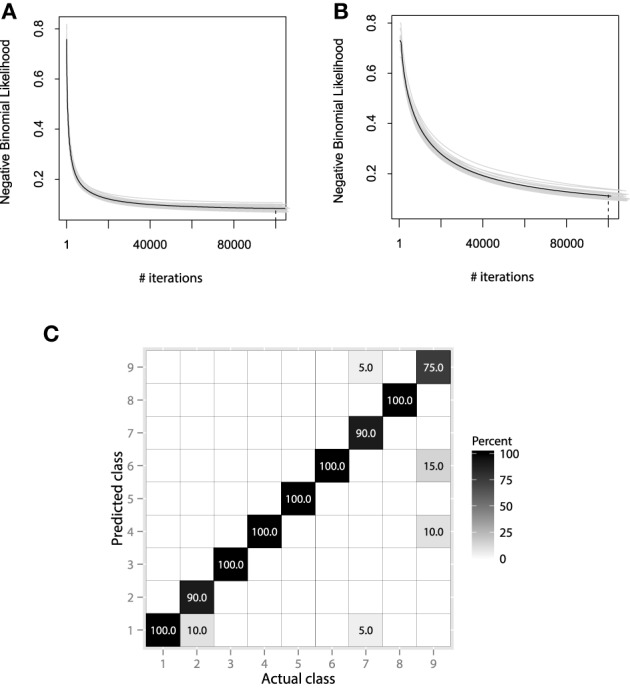
**(A)** Bootstrap estimates of *M* for class 1 of CNAE, held-out error with λ = 0.01. **(B)** Bootstrap estimates of *M* for class 1 of CNAE, held-out error with λ = 0.001. **(C)** Confusion matrix for the CNAE-9 test set.

The bootstrap estimates for the number of base-learners *M* of the GBM described above with λ = 0.01 are presented in Figure 15A. This chart represents the convergence rates for the GBM, fitted only for the first class, but similar pictures can be obtained for any of the other eight classes. We can deduce that although the held-out errors don't start growing with the number of iterations substantially increased, there might be no actual demand in this exceeding amount of learning.

The test set classification result from building the above mentioned model with all the 100,000 boosts reaches exactly 95%, or 171 correct out of 180. Reducing the number of iterations *M* by half, *M* = 50,000 leads to a slight decrease in test set accuracy to 94.44%.

Now we will arrange a similar simulation experiment with the same learning parameters, except with the shrinkage reduced to λ = 0.001. The resulting bootstrap estimates for the first class GBM are presented in Figure 15B.

Although the training error at the end of the learning process is higher than that of the previous experiment, the test set error remains at the same level with 95% correct classifications, which indicates the similar generalization properties of the model designed. In the previous works with other models, tested on this dataset, the maximal test set accuracy achieved was 92.78%, with the kNN classifier used on the dimensionality reduced to 200. The confusion matrix of the λ = 0.001 GBM on the test set is shown in Figure 15C. Values inside the boxes correspond to the percentage of the points of the actual class that have been assigned a chosen predicted class.

From the resulting confusion matrix we can see that the method works well on sparse data. If we analyze the confusion matrix, we can deduce that most of the errors come from the last 9th class. If we possessed the means to control the experiment and wanted to improve the accuracy of the deployed system, then we would have suggested investing more time into feature engineering to improve the predictions of that class.

#### 6.3.5. Application conclusion

We have successfully achieved an accurate result on the current application. But accuracy alone doesn't necessary imply anything about the fitted model behavior. Although we have built the overall resulting model from 9 one-class GBMs, each of the models relies on approximately 70 variables. The total number of the unique variables in the resulting 9-class boosted GLM model is 246. This is considerably sparse when compared to the original 856 dimensions, however, each of the classes relies on even lower dimensional sub-models. Given the original labels of the classes and variable names, one could also make a more detailed analysis of the low-dimensional variable interconnections between classifiers.

Together with the high-accuracy of the resulting model, we can conclude that this approach could easily and efficiently be adopted in the equivalent industrial application, not requiring any complex model design, just “off-the-shelf.”

## 7. Discussion

### 7.1. Research directions

There are two groups of promising neurorobotics applications for GBMs: the high-accuracy pattern recognition applications and the ensemble-based neural simulations. When considering pattern recognition problem, one can efficiently assess tasks like speech and motion recognition with boosted temporal models like HMM (Hu et al., 2007; Du et al., 2011). Another important application is the extraction of relevant information from large amounts of data. It is a general purpose problem, which has been efficiently solved with boosted ensemble models in the webpage ranking area (Burges et al., 2006; Clemencon and Vayatis, 2009). The same boosted ensemble ranking approach can be adopted in problems with the neural activity data (Lewickiy, 1998; Lotte et al., 2007).

In ensemble-based simulations, the main idea is to consider GBMs as the graph of submodels, where nodes are defined by base-learners and the edges are either shared parameters of base-learners (e.g., branch of the tree) or some calculated measure of similarity between base-learners [e.g., correlation of the residuals or the Kullback-Leibler divergence (Shibata, 1997; Runnalls, 2007)]. It is then feasible to involve graph formation techniques like preferential attachment and rewiring into the learning process in order to achieve different graph topologies. This would allow a flexible yet very natural way to simulate neural structures within the traditional pattern recognition problems. Based on different properties of the obtained graph (Bullmore and Sporns, 2009) one would be able to investigate properties of the resulting ensemble model, comparing it to the behavior of the real neural models (Latora and Marchiori, 2001; Li and Chen, 2003; Simard et al., 2005). Besides, graph representation of the ensemble models would allow one to visually examine the resulting models through graph visulization tools and layouts (Fruchterman and Reingold, 1991; Hu, 2005).

### 7.2. GBM drawbacks

Gradient boosting machines are a powerful method that can effectively capture complex non-linear function dependencies. This family of models has shown considerable success in various practical applications. Moreover the GBMs are extremely flexible and can easily be customized to different practical needs. However, all these results and benefits do not come for free. Although GBMs can be considered to be a methodological framework than a particular method, they still have several drawbacks.

The most noticeable problem of the GBMs that arises in practice is their memory-consumption. The cost of storing a predictive model depends on the number of boosting iterations used for learning. As we discussed in the regularization section, to reduce the effects of overfitting, the optimal number of iterations for a suitable shrinkage parameter can be considerably large. In some accuracy-intensive applications like intrusion detection systems, the desired number of iterations can easily be of the range of tens of thousands. Handling such massive models requires the storage of all the parameters of each of the fitted base-learners. This problem can be partially circumvented with the extensive usage of sparse base-learners or with the methods of the ensemble simplification (Chen et al., 2009; Kulkarni and Sinha, 2012). However, this problem with the memory consumption is common to all the ensemble methods and shows up more significantly with the increased number of models one chooses to store.

Another problem of GBM that naturally arises from the high memory-consumption is the evaluation speed. To use the fitted GBM model to obtain predictions, one has to evaluate all the base-learners in the ensemble. Despite the simplicity of each of the base-learners, when the ensemble is considerably large, obtaining predictions at a fast pace can become time-consuming. Therefore, using GBMs in intensive online tasks would most likely require the practitioner to accept a trade-off between the model complexity and the desired number of function evaluations per time interval. However, when the GBM ensemble is already learnt, one can take full advantage of parallelization to obtain the predictions.

Despite the parallelization of the function evaluation, the learning procedure is essentially sequential and has problems with parallelization by design. This is not a unique problem of GBMs, but unlike many other ensemble techniques like random forests, this makes them on average slower to learn. This issue can be partially alleviated using the mini-batch learning and other tricks to improve the computation costs of gradient-based learning (Cotter et al., 2011), however, the learning algorithm still relies on the previously learned fits, by design. A different approach to parallelization of the GBMs would be to parallelize each of the boosting iterations, which can still bring improvement in the evaluation speed.

The above mentioned problems are purely computational and thus can be considered the cost of using a stronger model. As we have described, GBMs are highly applicable, providing various useful properties to the practitioner. Moreover, as previously discussed, they allow for relatively easy result interpretation, thus providing the researcher with insights into the fitted model.

And as we previously noted, GBMs can be considered as a framework for model design, thus giving practitioners the opportunity not only to customize, but also to design very specific novel GBM models for particular tasks. This high flexibility has led to development of a wide range of GBM algorithms, both designed for different specific loss-functions and utilizing different data-specific base-learners.

Another shortcoming of the GBMs is that there is currently no fast and efficient model and implementation of the smooth continuous base-learner that capture interactions. As we have seen from the application examples, interactions between variables can play a crucial role in the particular predictive model design. However, only decision trees can efficiently capture non-trivial interactions between variables in reasonable computation time. It is yet worth noting, that batch exploitation of several base-learners can potentially neglect this problem, but such algorithms are currently not used in practice due to specificity in the GBM model design.

## 8. Conclusion

In this tutorial we have presented the methodology of the gradient boosting machines. Both the theoretical framework and the design options were described and illustrated. We have discussed all the essential stages of designing a particular model for one's practical needs. Interpretation issues have been addressed and presented as an essential part of the analysis.

The capabilities of the GBMs were investigated on a set of real-world practical applications. In every case, GBMs provided excellent results in terms of accuracy and generalization. In addition, the GBMs offered additional insights into the resulting model design, allowing for deeper investigation and analysis of the modeled effects.

### Conflict of interest statement

The authors declare that the research was conducted in the absence of any commercial or financial relationships that could be construed as a potential conflict of interest.
